# Detection and differentiation of *Burkholderia* species with pathogenic potential in environmental soil samples

**DOI:** 10.1371/journal.pone.0245175

**Published:** 2021-01-07

**Authors:** Sujintana Janesomboon, Veerachat Muangsombut, Varintip Srinon, Chatruthai Meethai, Chayada S. Tharinjaroen, Premjit Amornchai, Patoo Withatanung, Narisara Chantratita, Mark Mayo, Vanaporn Wuthiekanun, Bart J. Currie, Joanne M. Stevens, Sunee Korbsrisate

**Affiliations:** 1 Department of Immunology, Faculty of Medicine Siriraj Hospital, Mahidol University, Bangkok, Thailand; 2 Microbiology Laboratory, Veterinary Diagnostic Center, Faculty of Veterinary Science, Mahidol University, Nakhon Pathom, Thailand; 3 Division of Clinical Microbiology, Department of Medical Technology, Faculty of Associated Medical Sciences, Chiang Mai University, Chiang Mai, Thailand; 4 Infectious Diseases Research Unit, Faculty of Associated Medical Sciences, Chiang Mai University, Chiang Mai, Thailand; 5 Mahidol-Oxford Tropical Medicine Research Unit, Faculty of Tropical Medicine, Mahidol University, Bangkok, Thailand; 6 Department of Microbiology and Immunology, Faculty of Tropical Medicine, Mahidol University, Bangkok, Thailand; 7 Global and Tropical Health Division, Menzies School of Health Research, Charles Darwin University, Darwin, Northern Territory, Australia; 8 Department of Infectious Diseases, Royal Darwin Hospital, Darwin, Northern Territory, Australia; 9 The Roslin Institute and Royal (Dick) School of Veterinary Studies, University of Edinburgh, Edinburgh, Midlothian, United Kingdom; ENEA Centro Ricerche Casaccia, ITALY

## Abstract

The *Burkholderia pseudomallei* phylogenetic cluster includes *B*. *pseudomallei*, *B*. *mallei*, *B*. *thailandensis*, *B*. *oklahomensis*, *B*. *humptydooensis* and *B*. *singularis*. Regarded as the only pathogenic members of this group, *B*. *pseudomallei* and *B*. *mallei* cause the diseases melioidosis and glanders, respectively. Additionally, variant strains of *B*. *pseudomallei* and *B*. *thailandensis* exist that include the geographically restricted *B*. *pseudomallei* that express a *B*. *mallei*-like BimA protein (BPBM), and *B*. *thailandensis* that express a *B*. *pseudomallei*-like capsular polysaccharide (BTCV). To establish a PCR-based assay for the detection of pathogenic *Burkholderia* species or their variants, five PCR primers were designed to amplify species-specific sequences within the *bimA* (*B**urkholderia* intracellular motility A) gene. Our multiplex PCR assay could distinguish pathogenic *B*. *pseudomallei* and BPBM from the non-pathogenic *B*. *thailandensis* and the BTCV strains. A second singleplex PCR successfully discriminated the BTCV from *B*. *thailandensis*. Apart from *B*. *humptydooensis*, specificity testing against other *Burkholderia* spp., as well as other Gram-negative and Gram-positive bacteria produced a negative result. The detection limit of the multiplex PCR in soil samples artificially spiked with known quantities of *B*. *pseudomallei* and *B*. *thailandensis* were 5 and 6 CFU/g soil, respectively. Furthermore, comparison between standard bacterial culture and the multiplex PCR to detect *B*. *pseudomallei* from 34 soil samples, collected from an endemic area of melioidosis, showed high sensitivity and specificity. This robust, sensitive, and specific PCR assay will be a useful tool for epidemiological study of *B*. *pseudomallei* and closely related members with pathogenic potential in soil.

## Introduction

*Burkholderia pseudomallei*, an environmental Gram-negative bacillus, is the causative agent of melioidosis. This potentially fatal infectious disease affects an estimated 165,000 people worldwide, with an estimated 89,000 deaths (54% mortality) per year [1]. *B*. *pseudomallei* infections occur mainly through contact with wet contaminated soil or surface water where the bacteria is prevalent [2]. This bacterium forms part of a phylogenetic cluster of *Burkholderia* species including *B*. *mallei*, *B*. *thailandensis*, *B*. *humptydooensis*, *B*. *oklahomensis* and *B*. *singularis* [3, 4]. Notably only *B*. *pseudomallei* and *B*. *mallei* are associated with severe clinical disease with high mortality. As a highly evolved obligate pathogen with no environmental reservoir, *B*. *mallei* causes glanders in horses, donkeys and other solipeds, and less commonly zoonotic disease in humans [5].

*B*. *thailandensis*, which is a significantly less pathogenic species than *B*. *pseudomallei* with no associated biosecurity threat, is often found to coexist with *B*. *pseudomallei* as mixed populations in soil and water in Southeast Asia [6]. The prevalence of *B*. *pseudomallei* in soil and water defines geographic regions where humans and livestock are at risk of melioidosis. Recently, a survey of these *Burkholderia* species in soil samples from three regions of Thailand demonstrated that the ratio of *B*. *pseudomallei* to *B*. *thailandensis* in the Northeast and East, the areas of high prevalence of melioidosis, are much higher than the Central region [6]. Whilst *B*. *thailandensis* is commonly regarded as nonpathogenic, on rare occasions *B*. *thailandensis* infections in humans have been reported [7–9]. Thus, an effective and simple assay to differentiate between these two species would be clinically useful.

Recently, a phylogenetic subgroup distinct from the ancestral *B*. *thailandensis* population incorporating *B*. *thailandensis* variants harboring the *B*. *pseudomallei*-like polysaccharide, and known as *B*. *thailandensis* capsular variants (BTCV), were described [10]. To date BTCV have only been isolated from environmental soil or water in areas where melioidosis is endemic, notably in Thailand in 2018 [11] and Cambodia (strain E555) in 2010 [10]. There are a total of three case reports involving infection with a BTCV strain between 2003 and 2017 in the U.S. [9] and in China [7]. Remarkably, the BTCV possess several *B*. *pseudomallei*-like phenotypes including the presence of a *B*. *pseudomallei*-like capsule, resistance to human complement binding, and increased intracellular macrophage survival, although they were no more virulent in a murine infection model than a prototypic *B*. *thailandensis* strain [10]. Interestingly, immunization of mice with BTCV induced a significantly better protective immune response to *B*. *pseudomallei* challenge than that of prototypic *B*. *thailandensis*, indicating the importance of the *B*. *pseudomallei*-like capsule of the BTCV for immunogenic and protective efficiency [12]. In addition, Riyapa et al. reported that the BTCV strain E555 induced less ROS and neutrophil extracellular trap formation in PMNs than *B*. *thailandensis* E264, suggesting the importance of the *B*. *pseudomallei* capsule in evading the induction and killing activity of neutrophil extracellular trap formation [13]. Little is known about the distribution of the BTCV in the environment, and its association with the immune status of people living in melioidosis endemic areas. There is interest within the scientific community in the use of BTCV strains, instead of the prototypic *B*. *thailandensis* strains, as a surrogate for experimental studies involving *B*. *pseudomallei* [14, 15].

The other three members of the *Burkholderia pseudomallei* complex have been isolated from the environment in very restricted geographical locations, specifically *B*. *oklahomensis* from Oklahoma, US, *B*. *humptydooensis* from Humpty Doo in the Northern Territory of Australia and *B*. *singularis* from a water source in Australia [3, 4, 16]. *B*. *oklahomensis* was associated with a non-fatal pelvic wound infection in a farmer as a result of a tractor accident. Identical strains were isolated from the wound and the environment and shown to display significantly lower virulence in a Guinea pig model of infection [16], and more recently in murine and hamster melioidosis models [17]. *B*. *humptydooensis* was initially described as a *B*. *thailandensis*-like species able to assimilate arabinose in a similar manner to *B*. *thailandensis*, and has been named the fifth member of the *B*. *pseudomallei* complex [18, 19]. At present this microorganism has not been associated with any human disease and it is not possible to distinguish *B*. *humptydooensis* from closely related species by the commonly used biochemical and fatty acid methyl ester analysis [19]. The sixth member of the *Burkholderia pseudomallei* complex, *B*. *singularis*, was described by Vandamme *et al* in 2017 [4]. This micro-organism was isolated from a water source in Australia. *B*. *singularis* appears to be an opportunistic pathogen of Cystic Fibrosis (CF) patients, having been isolated from a CF patient in Germany and a patient in Canada [4].

Besides the existence of *B*. *thailandensis* and its capsule variants, genetic diversity within the *B*. *pseudomallei* strains also exists, where some geographically restricted strains express a *B*. *mallei*-like BimA (BPBM). Originally identified in a proportion of Australian *B*. *pseudomallei* clinical and environmental strains [20], these BPBMs have also been recorded in India [21]. BPBMs are implicated in neurological melioidosis in patients [22], and can only be distinguished from prototypic *B*. *pseudomallei* strains using molecular techniques. Although BPBMs have not yet been isolated in Southeast Asia, the possibility that this variant strain could be present in Thailand or other countries cannot be excluded. Taken together with BTCV, prototypic *B*. *pseudomallei* and BPBM strains represent an important group of microorganisms with pathogenic potential, whose environmental presence would be indicative of significant human and animal disease risk.

Conventional culture using Ashdown’s selective agar has remained the “gold standard” for detection of *B*. *pseudomallei*, *B*. *thailandensis* and other related species from environmental and clinical specimens. However, it is recognized that this method is limited by the length of time to culture these organisms, and the similarities in appearance of colony morphology make it redundant in the differentiation of *B*. *pseudomallei* from *B*. *thailandensis* [3, 23]. Additional assays are required to differentiate them. Therefore, singleplex PCR techniques targeting a variety of different genes for example the T3SS1 [24] and a serine metalloprotease (*mprA*) [25], have been established for detection of *B*. *pseudomallei*. A multiplex PCR targeting the *B*. *pseudomallei* specific gene encoding a Tat domain protein (BPSS0658), a *B*. *thailandensis*-specific gene (BTH_I1515) and a conserved *B*. *cenocepacia* gene (BCAM2834) has been demonstrated to detect *B*. *pseudomallei*, *B*. *thailandensis* and members of the *B*. *cepacia* complex in both soil and clinical samples [26]. However, the current PCR-based techniques described to date cannot detect the BTCV or differentiate *B*. *pseudomallei* from BPBM. Thus, a simple and sensitive PCR assay to identify members of the *B*. *pseudomallei* complex with pathogenic potential in environmental soil samples is required.

In this study, we developed a highly sensitive and specific multiplex PCR-based method for screening for the presence of *B*. *pseudomallei*, *B*. *thailandensis* and their variant strains with pathogenic potential in soil samples. An additional simplex PCR enabled discrimination between *B*. *thailandensis* and BTCV strains. The target DNA for amplification in these assays is the *bimA* (*B**urkholderia* intracellular motility A) gene, which is a bacterial virulence factor that contributes to *B*. *pseudomallei* and *B*. *thailandensis* actin-based motility in infected host cells [27, 28]. This robust PCR method was successful in the discrimination of the different *Burkholderia* species and variant strains tested, and in spiked soil samples. This method will be an invaluable tool for epidemiological studies in melioidosis endemic and non-endemic areas.

## Materials and methods

### Ethical considerations

All clinical strains used in our study were collected as part of previous clinical studies with approval from the relevant Research Ethics committees, patient consent where required and de-identified before use in this work. All soil and water sample collections which took place on the private land were conducted after receiving permission from the owners.

### Bacterial strains and DNA isolation

The bacterial strains used in this study are listed in Table 1. *Burkholderia* spp. DNA, including *B*. *pseudomallei* (35 isolates), *B*. *pseudomallei* (BPBM) (20 isolates), *B*. *thailandensis* (30 isolates), BTCV (10 isolates), *B*. *mallei* (2 isolates), *B*. *cepacia* (7 isolates), *B*. *multivorans* (3 isolates), *B*. *ubonensis* (3 isolates), *B*. *anthina* (2 isolates), *B*. *cenocepacia* (2 isolates), *B*. *diffusa* (2 isolates), *B*. *humptydooensis* (2 isolates) *B*. *territorii* (2 isolates), *B*. *pseudomultivorans* (1 isolate), *B*. *oklahomensis* (1 isolate) and *B*. *vietnamiensis* (1 isolate) were used in this study. *Burkholderia* spp. including, *B*. *thailandensis*, BTCV, *B*. *cepacia*, *B*. *multivorans*, *B*. *oklahomensis* and *B*. *vietnamiensis* were cultured in Luria-Bertani broth (LB; Titan Biotech Ltd, Rajasthan, India). Strains of other bacterial species used to determine the specificity of the multiplex PCR were *Pseudomonas aeruginosa*, *Escherichia coli*, *Acinetobacter baumannii*, and *Staphylococcus aureus*. These bacteria were also cultured in Luria-Bertani (LB) broth at 37°C for 24 h.

**Table 1:** List of bacterial isolates used in this study.

Bacteria	Strains/ isolates	Sequence type	Source/ location	Reference
***Burkholderia* spp.**				
*B*. *pseudomallei*	576a	501	Human/Sunpasitthiprasong Hospital, Ubon Ratchathani province, Thailand	[29, 30]
	1026b	102		
	1106a	70		
	1710a	177		
	K96243	10		
	NCTC10276	46	Human/ Bangladesh	[31]
	956a, H2613a, H2659a, H2660a	54	Human/ Sunpasitthiprasong Hospital, Ubon Ratchathani province, Thailand	[32]
	1986a, H2644a, H2677a, H2689b	70		
	2396a	58		
	H1248a	298		
	H2708a, H2820a	60		
	E0024, E0411	54	Soil/ Thailand	[11]
	E0031, E0383	60		
	E0358, E0359	70		
	EFT01, EFT02, EFT03, EFT04, EFT05, EFT06, EFT07, EFT08, EFT09, EFT10, EFT11	-		
*B*. *pseudomallei* expresses a *B*. *mallei*-like *bimA*	MSHR33	647	Human/Royal Darwin Hospital, Darwin, Northern Territory, Australia	[20, 33]
	MSHR491	126		
	MSHR668	129		
	MSHR1790	439		
	MSHR2262	435		
	MSHR2375	439		
	MSHR2585	778		
	MSHR3325	118		
	MSHR3326	734		
	MSHR3448	680		
	MSHR3509	259		
	MSHR3522	809		
	MSHR3677	456		
	MSHR3689	809		
	MSHR3739	838		
	MSHR3835	778		
	MSHR3902	844		
	MSHR4176	853		
	MSHR4238	869		
	MSHR4445	886		
*B*. *thailandensis*	D1, DV1	-	Soil/ Thailand	[34]
* *	E264	80	Soil/ Thailand	[35]
	DW503	80		
	E27	74	Soil/ Thailand	[11]
	E327	77		
	E152, E153, E154, E158, E159, E169, E173, E174, E175, E177, E201, E202, E205, E207, E421, E426, E427, E430, E433, E435, E436, E438, E440, E441	-		
*B*. *thailandensis* expresses a *B*. *pseudomallei*-like capsule	E555	696	Soil/ Cambodia	[10]
	SBXSR001, SBXSR007, SBXPL001, SBXPL015, SBXRY031, SBXPR001, SBXCC001, SBXCC003	696	Soil/ Thailand	[11]
	WBXUBA33005104	696	Water/ Thailand	[11]
*B*. *mallei*	NCTC 3709	40	Animal/ India	[36]
	NCTC 12938	40	Animal/ China	
*B*. *cepacia*	U668, 10223	-	Human/ Thailand	[32]
	NCTC10743, NCTC10744		Human/ US	[36, 37]
	ATCC 25416	10	Soil/ US	[37]
	MSMB591	1581	Soil/ Australia	[36]
	MSMB1184	1615		
*B*. *multivorans*	LMG16660	899	Human/ UK	[38]
	MSMB2008	1767	Soil/ Australia	[36]
	MSMB2021	1768		
*B*. *ubonensis*	DMST886	-	Soil/ Thailand	National Institute of Health, Thailand
	MSMB22	1129	Soil/ Australia	[36, 39]
	MSMB1162	1171		
*B*. *anthina*	MSMB649, MSMB1506	-	Soil/ Australia	[36]
*B*. *cenocepacia*	MSMB364	429	Water/ Australia	[36]
	MSMB384	320		
*B*. *diffusa*	MSMB375	131	Water/ Australia	[36]
	MSMB583	464		
*B*. *humptydooensis*	MSMB1588	1441	Soil/ Australia	[36]
	MSMB43	786	Water/ Australia	[3]
*B*. *territorii*	MSMB599	723	Soil/ Australia	[36]
	MSMB793			
*B*. *oklahomensis*	NCTC 13387	81	Human/ US	[40]
*B*. *pseudomulti- vorans*	MSMB2199	-	Soil/ Australia	[36]
*B*. *vietnamiensis*	LMG6999	524	Human/ Vietnam	[38]
**Non-*Burkholderia* spp.**				
*A*. *baumannii*	ATCC 19606	-	Human/ US	[41]
	No.9, No. 40, No. 72	-	Sewage/ Thailand	This study
*E*. *coli*	Ec1, Ec2, Ec3, Ec4, Ec5	-	Sewage/ Thailand	This study
*P*. *aeruginosa*	ATCC27853	-	Human/ US	[42]
	BF311, P256, P338, P362, SP5, SP749, SP770, SP780/2, U1466	-	Sewage/ Thailand	This study
*S*. *aureus*	ATCC25923	-	Human/ US	[43]
	Staph04, Staph05, Staph40, Staph156	-	Food/ Thailand	This study

(-), not available.

All of the *Burkholderia* isolates were collected from previous study (Table 1). Clinical isolates of *B*. *pseudomallei* from human were obtained from Sunpasitthiprasong Hospital, Ubon Ratchathani province, Thailand and Royal Darwin Hospital, Darwin, Northern Territory, Australia. Sources of the *Burkholderia* and non-*Burkholderia* isolates as well as their Multi Locus Sequence Type (MLST) (where known) are shown in Table 1.

Bacterial genomic DNA was extracted from 1 ml of bacteria cultured in LB broth overnight at 37°C using a Genomic DNA Mini Kit (Geneaid Biotech Ltd., New Taipei City, Taiwan) according to the manufacturer’s instructions. DNA extracts were stored at -20°C. Alternatively, a colony boiling method was used in which a loopful of bacteria cultured on LB agar was suspended in 100 μl sterile distilled water, followed by heat inactivation at 99°C for 15 min. One microliter of DNA from each Genomic DNA extraction was used for PCR amplification.

### PCR primers targeting *Burkholderia bimA* sequences

The *bimA* sequences of *B*. *mallei* (ATCC 23344), *B*. *pseudomallei* (K96243), and *B*. *thailandensis* (E264) from the National Center for Biotechnology Information (NCBI) were aligned to design PCR primers using Primer-BLAST service tools (https://www.ncbi.nlm.nih.gov/) with default parameters. The structures of the selected primers were evaluated by Oligo Analyzer 3.1 (https://sg.idtdna.com/calc/analyzer), and synthesized by Integrated DNA Technologies, Inc. (Coralville, IA, USA).

### Multiplex PCR conditions

*B*. *pseudomallei* (K96243), *B*. *thailandensis* (E264), and *B*. *mallei* (NCTC12938) genomic DNA were used as DNA templates for optimization of PCR conditions. Multiplex PCR detection was performed in a total volume of 25 μl containing 1 μl of bacterial lysates or purified genomic DNA, 0.2 mM of dNTPs, 1× of Q5 reaction buffer, 1× of Q5 high GC enhancer, 0.02 units of Q5 high-fidelity DNA polymerase (New England Biolabs, Inc., MA, USA), five PCR primers including 0.92 μM of BimA_com_-R primer, 0.72 μM of BimA_Bps_-F primer, 0.10 μM of BimA_BPBM_-F primer and 0.30 μM each of BimA_Bth_-F and BimA_Bth_-R primers.

The DNA amplification involved initial denaturation at 98°C for 3 min, and 35 cycles at 98°C for 30 s, 68°C for 45 s, and 72°C for 30 s, followed by a final extension for 10 min. The PCR products were verified by 1.5% agarose gel electrophoresis (Vivantis Technologies Sdn. Bhd., Selangor Darul Ehsan, Malaysia) and visualized using a UV transilluminator (Syngene, Cambridge, UK). A GeneRuler 100 bp Plus DNA Ladder (Thermo Fisher Scientific, Inc., MA, USA) was included as a DNA marker.

### Singleplex PCR to differentiate *B*. *thailandensis* from the BTCV

PCR detection was performed in a total volume of 25 μl containing 1 μl purified genomic DNA, 0.2 mM of dNTPs, 1× of Q5 reaction buffer, 1× of Q5 high GC enhancer, 0.02 units of Q5 high-fidelity DNA polymerase (New England Biolabs, MA, US), and 0.4 μM of each BimA_Bth_-2F and BimA_Bth_-R primers. The DNA amplification involved initial denaturation at 98°C for 3 min, and 35 cycles at 98°C for 30 s, 57°C for 30 s, and 72°C for 30 s, followed by a final extension for 10 min. PCR products were visualized following 1.5% agarose gel electrophoresis (Vivantis Technologies Sdn. Bhd., Malaysia).

### Detection of *B*. *pseudomallei* and *B*. *thailandensis* from spiked soil samples

Control soil lacking detectable *B*. *pseudomallei* and *B*. *thailandensis* by PCR targeting the 16S rRNA gene, were used for extraction and sensitivity testing. Overnight cultures of *B*. *pseudomallei* K96243, *B*. *pseudomallei* MSHR668 and *B*. *thailandensis* E555 were adjusted to 0.5 McFarlane with 1× phosphate-buffered saline (PBS). The number of viable bacteria was determined by plating the serial dilution of bacterial culture on LB agar. Bacterial suspensions were serially 10-fold diluted with 1× PBS to approximately 10−10^3^ CFU/ml, and then 100 μl of each bacterium suspension was inoculated into 20 g soil to achieve 1–100 CFU/20 ml. The inoculated soil samples were incubated in 20 ml of Ashdown’s broth at 37°C for 24 h before DNA extraction using a DNeasy PowerSoil Kit (Qiagen, Hilden, Germany). The final precipitated DNA preparation was eluted with 20 μl of elution buffer (Qiagen, Hilden, Germany) or nuclease-free water before amplification by the developed multiplex or singleplex PCR.

### Soil sample testing

For analysis of the environmental samples, 34 soil samples were collected from a rice field in a highly endemic area in Ubon Ratchathani (12 samples) and Khon Kaen (22 samples) provinces, Northeast of Thailand. These two provinces are 282 km apart. Soil samples were collected as previously described [44]. Essentially, 20 g of soil at 30 cm depth was collected and cultured for *B*. *pseudomallei*, *B*. *thailandensis* and BTCV by standard culture method. All of the soil samples were subjected to DNA extraction using a DNeasy PowerSoil Kit (Qiagen, Hilden, Germany) as described above. The extracted DNA samples were subjected to the multiplex PCR assay described above. *E*. *coli* 16S rRNA gene was amplified using the 27F and 518R primers to generate a DNA fragment of 527 bps [45]. These primers were included as a control following extraction from the soil samples.

## Results

### Design of PCR primers targeting *bimA* sequences for specific detection of *B*. *pseudomallei*, BPBM, *B*. *thailandensis* and BTCV

**Fig 1:**
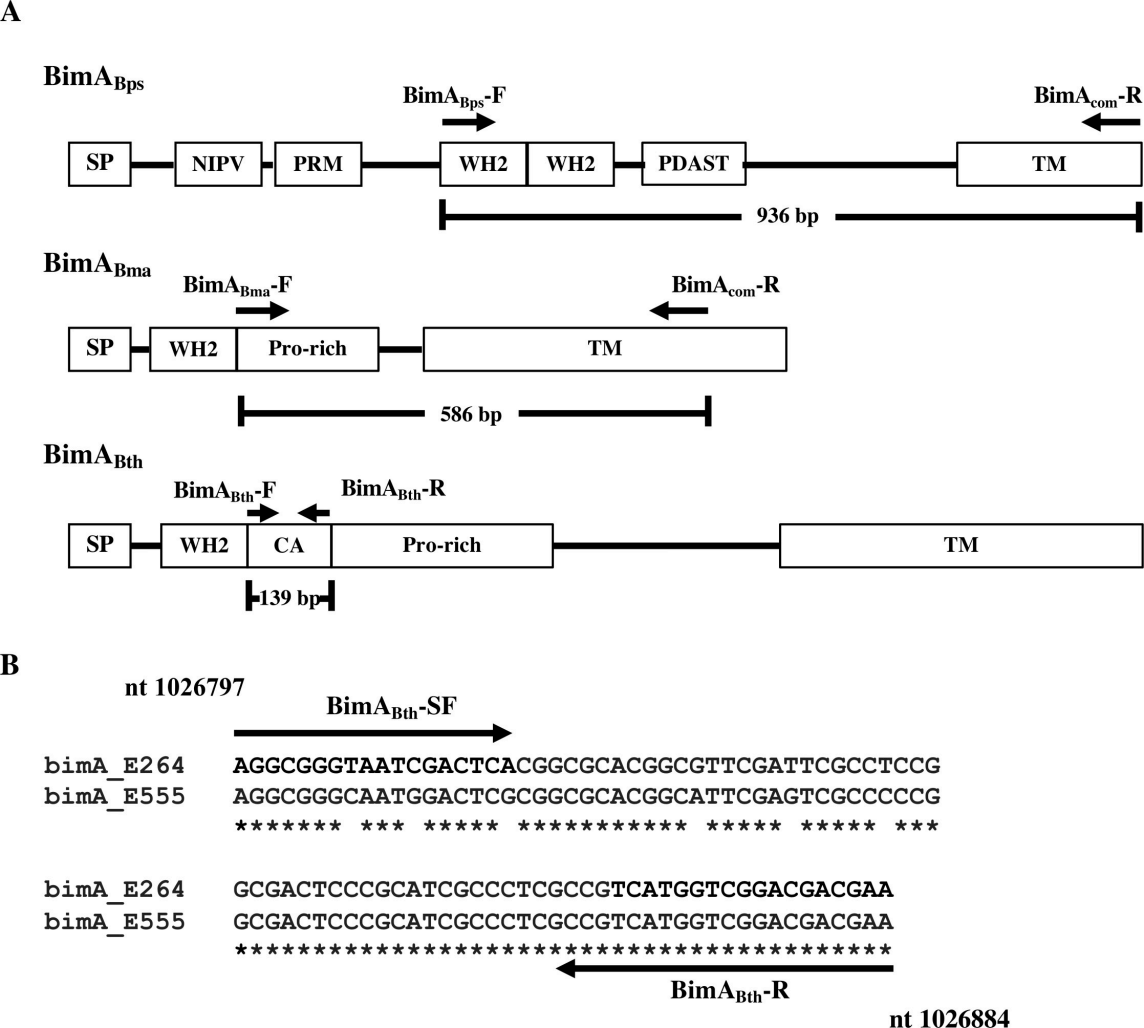
Schematic locations of PCR primers on various domains of *Burkholderia bimA* genes. (A) Locations of the multiplex PCR primers (BimA_Bps_-F/BimA_com-_R, Bim_BPBM_-F/BimA_com-_R, and BimA_Bth_-F/BimA_Bth-_R) for generating 963 bp, 586 bp, and 139 bp amplicons specific to *B*. *pseudomallei*, BPBM/ *B*. *mallei*, and *B*. *thailandensis*/ BTCV, respectively. (B) Locations of simplex PCR primers (BimA_Bth_-SF and BimA_Bth_-SR) on central and acidic (CA) domain of *bimA* gene for differentiation between typical *B*. *thailandensis* (strain E264) and BTCV (strain E555). Numbers refer to nucleotide positions on *B*. *thailandensis* E264 chromosome 2.

The *bimA* gene, that contributes to *B*. *pseudomallei* and *B*. *thailandensis* actin-based motility in infected host cells [46], varies in sequence specifically in the region of the gene encoding the extracellular actin-binding portion of the protein [27, 46, 47]. We chose to use this information to design PCR primers to discriminate between members of the *Burkholderia pseudomallei* complex, especially those with known or pathogenic potential. *In silico* analysis of prototypic *Burkholderia bimA* genes of *B*. *pseudomallei* K96243 (NC_006351), *B*. *thailandensis* E264 (NC_007651), and *B*. *mallei* ATCC23344 (NC_006349) revealed 5 oligonucleotides of which could be used in a multiplex PCR (Fig 1A). The BimA_Bps_-F and BimA_com_-R primers were designed to amplify 963 bp *bimA* amplicons of *B*. *pseudomallei*, whereas BimA_Bth_-F/BimA_Bth_-R and Bim_BPBM_-F/BimA_com_-R primers could amplify 139 bp and 586 bp *bimA* DNA fragments of the *bimA* genes of *B*. *thailandensis* and BPBM, respectively (Table 2). Since *B*. *mallei* is unable to survive in the environment, any *B*. *mallei*-like *bimA* amplicons in this assay are most likely to derive from a BPBM strain.

**Table 2:** Oligonucleotide primers and the size of *bimA* amplicons for detection of *B*. *pseudomallei*, *B*. *thailandensis*, *B*. *mallei* and their variants including BTCV and BPBM.

Primer name	Sequences 5′ to 3′	Size of amplicon	For detection of	References
BimA_Bps_-F	GATCGCTGAAGAAAAATCCG	963 bp	*B*. *pseudomallei*	This study
BimA_com_-R	CCTTGAGGTTTTCGTTGATG			
BimA_BPBM_-F	ATTCCTAACGCGACACCAAC	586 bp	BPBM and *B*. *mallei*	This study
BimA_com_-R	CCTTGAGGTTTTCGTTGATG			
BimA_Bth_-F	ATCCGAACGAAACACGCG	139 bp	*B*. *thailandensis* and BTCV	This study
BimA_Bth_-R	TTCGTCGTCCGACCATGA			
BimA_Bth_-SF	AGGCGGGTAATCGACTCA	87 bp	*B*. *thailandensis*	This study and [48]
BimA_Bth_-R	TTCGTCGTCCGACCATGA			
27F	AGAGTTTGATCMTGGCTCAG	527 bp	*E*. *coli*	[45]
518R	ATTACCGCGGCTGCTGG		16S rRNA	

A caveat of the multiplex PCR was the inability to differentiate between *B*. *thailandensis* and the BTCV since a 139 bp amplicon would be amplified from both. To aid in the design of primers for differentiating these *bimA* genes, we sequenced the 139 bp *bimA* amplicons from BTCV strain E555 and *B*. *thailandensis* strain E264 to identify sequence differences which could be used to design primers for use in an additional simplex PCR reaction (Fig 1B). In combination with primer BimA_Bth_-R, *bimA* PCR primer BimA_Bth_-SF based on central and acidic (CA) domain of *bimA* gene (corresponding to nucleotide positions 1,026,797–1,026,815 of *B*. *thailandensis* E264, Fig 1A), would lead to amplification of an 87 bp *bimA* DNA fragment from *B*. *thailandensis* strains but not from BTCV strains (Table 2).

### Validation of the *bimA* primers for detection of *B*. *pseudomallei*, *B*. *thailandensis* and their variants in multiplex and singleplex PCR assays

**Fig 2:**
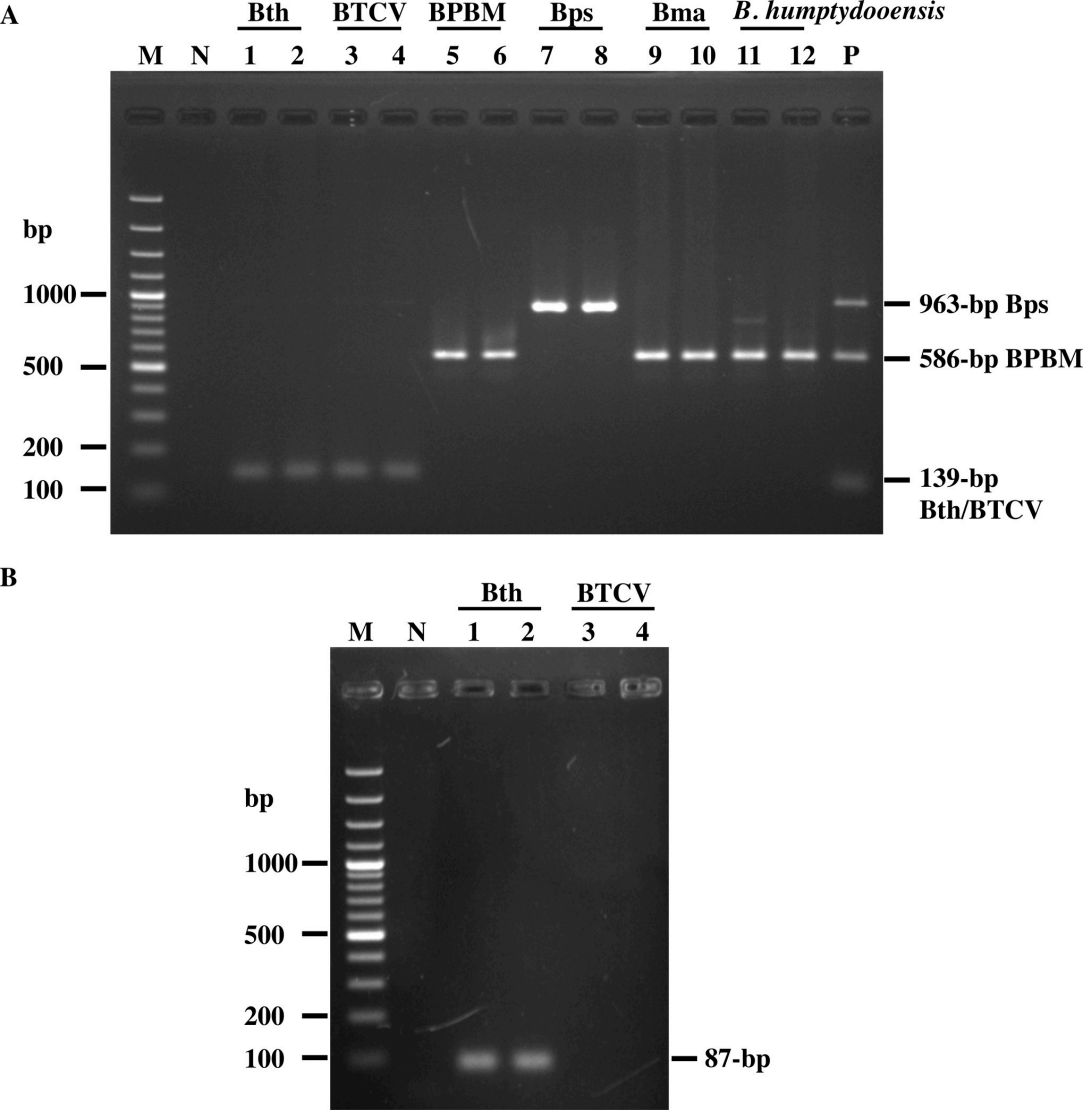
Agarose gel electrophoretic analysis of amplified DNA fragments generated from multiplex and singleplex PCR assays. (A) Multiplex PCR amplification of DNA templates from *B*. *thailandensis* strains E264, DV1 (lanes 1–2), the BTCV strains E555, SBXPR001 (lanes 3–4), BPBM strains MSHR3326, MSHR4445 (lanes 5–6), *B*. *pseudomallei* strains K92643, 1026b (lanes 7–8), *B*. *mallei* strains NCTC3709, NCTC12938 (lanes 9–10), and *B*. *humptydooensis* strains MSMB43, MSMB1588 (lanes 11–12). (B) Singleplex PCR amplification of *B*. *thailandensis* strains E264, DV1 (Bth; lanes 1–2) and *B*. *thailandensis* strain E555, SBXPR001 (BTCV; lanes 3–4). Lanes P and N are positive (mixture of *Burkholderia* spp. DNA) and negative (distilled water) controls, respectively. Lane M is 100 bp DNA ladder.

Genomic DNA from *B*. *pseudomallei*, *B*. *thailandensis*, *B*. *mallei* and their variant strains were mixed, and subjected to multiplex PCR with the 5 primers BimA_Bps_-F, BimA_com_-R, BimA_Bth_-F BimA_Bth_-R and Bim_BPBM_-F. Fig 2A shows that the *bimA-*specific primers can distinguish *B*. *pseudomallei* from *B*. *thailandensis* and BPBM strains *via* the generation of distinctive 963 bp, 139 bp, and 586 bp DNA fragments, respectively. Notably, *B*. *thailandensis* could not be differentiated from BTCV as well as *B*. *mallei* could not be differentiated from BPBM as they showed the identical amplicons at 139 and 586 bp, respectively.

To differentiate *B*. *thailandensis* from the BTCV strains, a singleplex PCR was established using primers BimA_Bth_-SF and BimA_Bth_-R. As demonstrated in Fig 2B, an 87 bp amplicon was obtained from *B*. *thailandensis* strain E264, but not from BTCV strain E555. Ten further BTCV isolates were tested in this assay, and whilst DNA amplicons of 139 bp were obtained in the multiplex PCR, no PCR products were generated in the singleplex *B*. *thailandensis*-specific PCR assay (S1 Fig). These findings indicate the successful combination of a multiplex and singleplex PCR to identify the BTCV.

In the Northern territory of Australia, a further member of the *B*. *pseudomallei* complex known as *B*. *humptydooensis* has been isolated from the environment [18]. This microorganism has not been associated with human or animal disease and has only been isolated from a restricted geographical area [19]. When genomic DNA from two *B*. *humptydooensis* strains (MSMB43 and MSMB1588) were included in the multiplex PCR, amplicons identical to the 586 bp amplicon of *B*. *mallei*/ BPBM *bimA* gene were seen (Fig 2A). DNA sequencing confirmed that the 586 bp PCR product amplified from *B*. *humptydooensis* was indeed *bimA*, demonstrating 99.05% homology to *B*. *humptydooensis* MSMB43 genome sequence in the NCBI database (GenBank No. CP013382; region 974821–975982). Comparisons of the *B*. *humptydooensis* (MSMB43 and MSMB1588) *bimA* sequence with that of *B*. *mallei* ATCC23344, *B*. *pseudomallei* MSHR491 (BPBM), *B*. *thailandensis* E264, *B*. *pseudomallei* K92643 and *B*. *singularis* LMG28154 demonstrated 94.03%, 93.52%, 85.89%, 84.68% and 0% nucleotide sequence homology, respectively. The variation within *B*. *humptydooensis* MSMB43 and MSMB1588 is 95.01% and 91.08% of gene homology and amino acid homology, respectively. Thus, it can be concluded that the *B*. *humptydooensis bimA* gene shows the greatest nucleotide sequence homology with *B*. *mallei* and BPBM *bimA* than with *B*. *thailandensis* or *B*. *pseudomallei* (S2 Fig). Whilst *B*. *humptydooensis* and BPBM could be detected but not differentiated by our PCR assay, we could use a second biochemical test to differentiate between the two since as *B*. *humptydooensis* can assimilate arabinose [19] whereas BPBM cannot.

### Specificity and sensitivity of multiplex PCR for detection of *B*. *pseudomallei* and *B*. *thailandensis*

To investigate the specificity of the *bimA* PCR primers, amplification of purified genomic DNA from *Burkholderia* spp. and other related bacterial species were undertaken. All *B*. *pseudomallei* (n = 35), *B*. *thailandensis* (n = 30), BTCV (n = 10), and BPBM (n = 20) produced PCR amplicons at the expected DNA fragment lengths. Similar to BPBM, all 2 isolates of *B*. *mallei* (NCTC3709, and NCTC12938) and 2 isolates of *B*. *humptydooensis* (MSMB43 and MSMB1588) led to amplification of products of the same size, reinforcing the data in Fig 2 that they could be detected, but could not be differentiated by the multiplex PCR and as predicted from Table 3. Analysis of the sequence type (ST) of each *Burkholderia* strain included in this study (Table 3) demonstrated that they belonged to multiple different ST, suggesting that our developed assay is not restricted to a specific ST. In addition, we have also BLASTed our primers to genomic DNA sequences of *B*. *singularis* (annotated contigs accession number FXAN01000001-FXAN01000135) and found no significant homology. Therefore, our assay is unlikely to be able to identify strains from this rare species.

**Table 3:** Specificity of the multiplex PCR assay against 121 isolates of *Burkholderia* spp. and 24 isolates of Gram-positive and Gram-negative bacteria.

Bacteria	Source [References]	No. of isolates	No. of PCR positive/ Total No. of isolates (%)
***Burkholderia* spp.**			
*B*. *pseudomallei*	Clinical [29, 30, 32, 49–52]	18	9/9 (100%)
	Environment [11]	17	24/24 (100%)
BPBM^a^	Clinical [20, 33]	20	20/20 (100%)
*B*. *thailandensis*	Environment [10, 11, 34]	30	30/30 (100%)
BTCV^b^	Environment [11]	10	10/10 (100%)
*B*. *mallei*	Animal [53]	2	2/2 (100%)
*B*. *humptydooensis*	Environment [3, 36]	2	2/2 (100%)
*B*. *multivorans*	Environment [38, 54]	3	0/3 (0%)
*B*. *ubonensis*	Environment [36, 39, 55]	3	0/3 (0%)
*B*. *anthina*	Environment [36]	2	0/2 (0%)
*B*. *cepacia*	Clinical [36, 37]	2	0/2 (0%)
	Environment [36, 56]	5	0/5 (0%)
*B*. *cenocepacia*	Environment [36]	2	0/2 (0%)
*B*. *diffusa*	Environment [36]	2	0/2 (0%)
*B*. *territorii*	Environment [36]	2	0/2 (0%)
*B*. *pseudomultivorans*	Environment [36]	1	0/1 (0%)
*B*. *oklahomemsis*	Clinical [40]	1	0/1 (0%)
*B*. *vietnamiensis*	Clinical [38, 55]	1	0/1 (0%)
**Non-*Burkholderia* spp.**			
*S*. *aureus*	Clinical	1	0/1 (0%)
	Food	4	0/4 (0%)
*P*. *aeruginosa*	Environment	10	0/10 (0%)
*E*. *coli*	Environment	5	0/5 (0%)
*A*. *baumannii*	Environment	4	0/4 (0%)

^a^*B*. *pseudomallei* that expresses a *B*. *mallei*-like *bimA;* ^b^*B*. *thailandensis* that expresses a *B*. *pseudomallei*-like capsule.

In contrast to *B*. *pseudomallei*, *B*. *thailandensis*, and their variant strains, no amplicons were generated when testing against *Burkholderia anthina*, *B*. *cepacia*, *B*. *cenocepacia*, *B*. *diffusa*, *B*. *multivorans*, *B*. *oklahomensis*, *B*. *pseudomultivorans*, *B*. *vietnamiensis*, *B*. *ubonensis*, and other unrelated bacterial species, including *Pseudomonas aeruginosa*, *Escherichia coli*, *Acinetobacter baumannii* and *Staphylococcus aureus* (Table 3). These results validate the specificity of this multiplex PCR assay for detection of *Burkholderia* species with known virulence or pathogenic potential based on the known genetic variation in the *bimA* genes.

**Fig 3:**
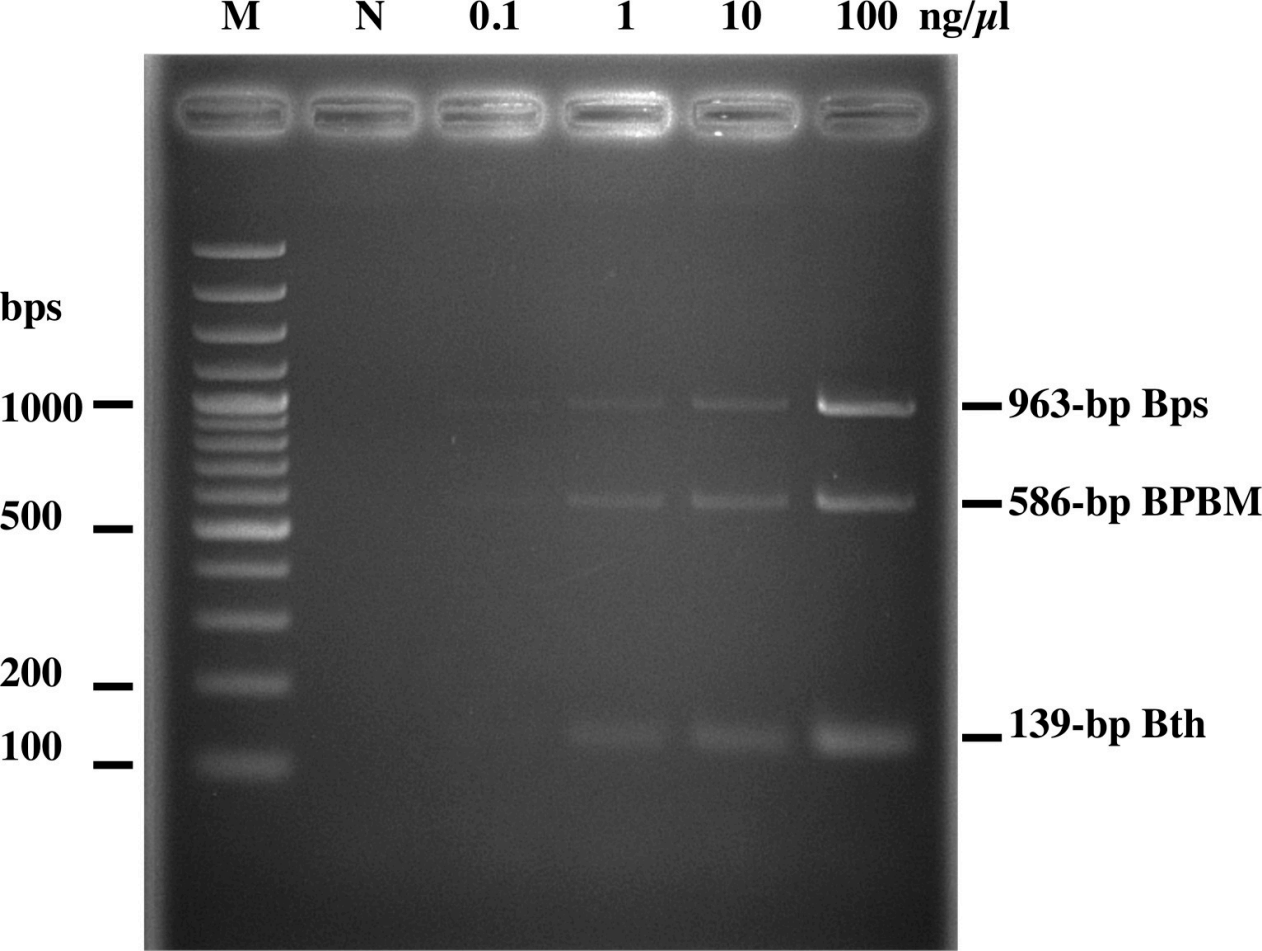
Sensitivity of multiplex PCR for detection of the *Burkholderia* spp. in a mixture of genomic DNA. Various concentrations (1, 10, and 100 ng/μl) of each *Burkholderia* genomic DNA were subjected to multiplex PCR including *B*. *pseudomallei* K92643 (Bps; 963 bp), *B*. *pseudomallei* MSHR491 (BPBM; 586 bp), and *B*. *thailandensis* E264 (Bth; 139 bp). Lanes M and N represent 100 bp DNA ladder and negative control (distilled water), respectively.

To investigate the sensitivity of the multiplex PCR, genomic DNA extracts from *B*. *pseudomallei*, *B*. *thailandensis*, and *B*. *mallei* were 10-fold serially diluted from 100 to 0.1 ng/μl and used as a template for the multiplex PCR. As shown in Fig 3, the sensitivity of the multiplex PCR for detecting extracted bacterial DNA is approximately 0.1–1.0 ng/μl. The sensitivity of the assay for *B*. *pseudomallei* and BPBM is around 0.1 ng/μl, and approximately 1 ng/μl for *B*. *thailandensis* (Fig 3).

**Fig 4:**
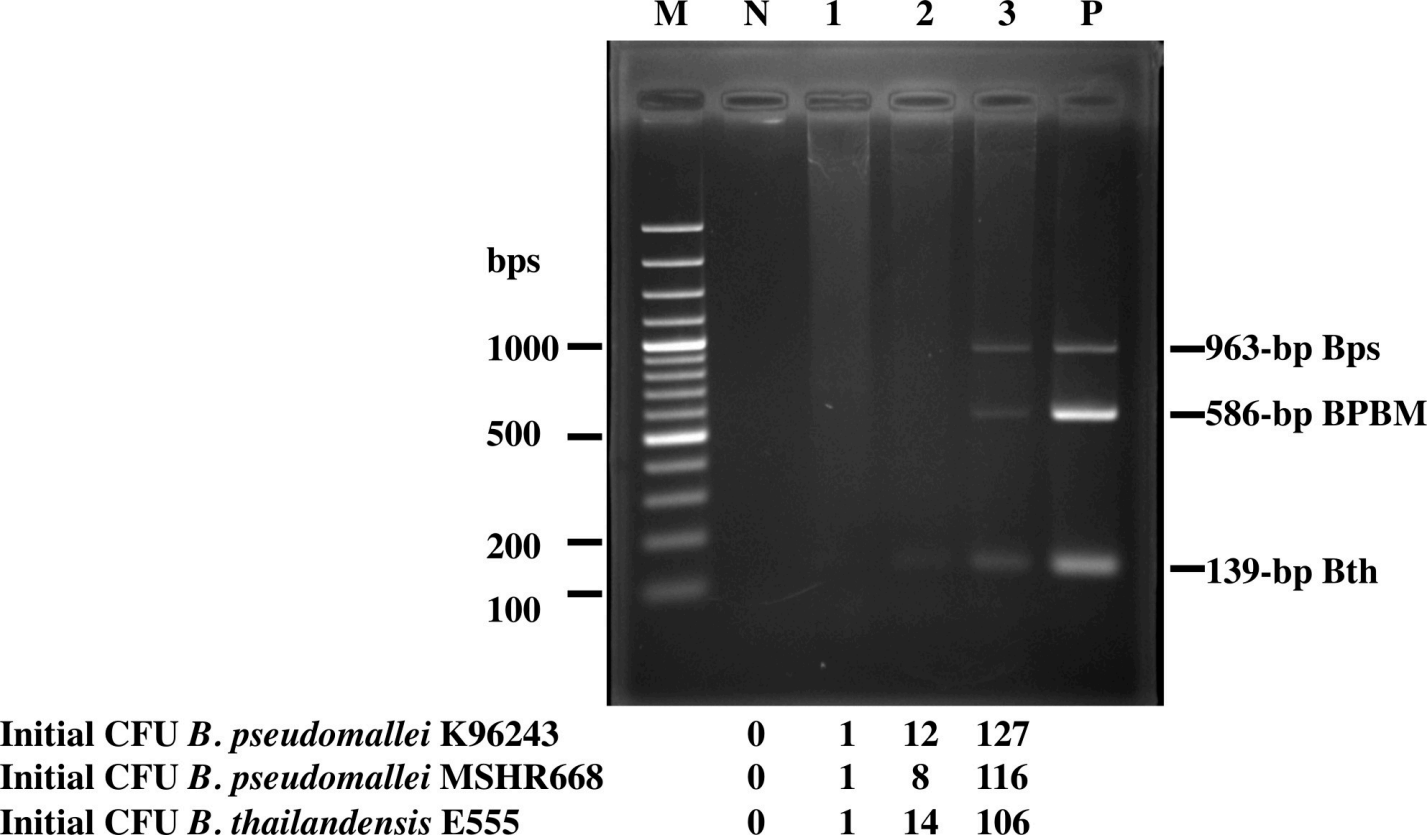
Multiplex PCR assay to detect *B*. *pseudomallei*, BPBM and *B*. *thailandensis* in spiked soil samples. Ten-fold serial dilution (lanes 1–3) of *B*. *pseudomallei*, BPBM and *B*. *thailandensis* were spiked into 20 g soil sample before DNA extraction and multiplex PCR. Lanes P (mixture of *Burkholderia* spp. genomic DNA) and N (no added bacteria) are positive and negative controls, respectively. Lane M is 100 bp DNA ladder.

To detect *B*. *pseudomallei* and *B*. *thailandensis* in soil samples, we next tested the ability of our PCR system to detect *Burkholderia* spp. in spiked soil samples. A crucial step of bacterial enrichment is performed on the first day of our protocol, which is then followed by an effective method for DNA extraction and finally PCR amplification. Twenty grams of sterile soil were spiked with known colony forming unit (CFU) of *B*. *pseudomallei*, a BPBM strain and *B*. *thailandensis*, added to 20 ml of Ashdown’s broth, and incubated at 37°C for 24 h with shaking. Next, DNA was extracted and subjected to multiplex PCR. As shown in Fig 4, we obtained the three expected DNA fragments of 963, 139, and 586 bp corresponding to *B*. *pseudomallei*, *B*. *thailandensis*, and BPBM, respectively. The limit of detection of this assay in an inoculated soil sample was as low as 127, 106 and 116 CFU/20 g soil, respectively, which is equivalent to approximately 6, 5, and 6 CFU/g of soil sample.

### Comparison of the sensitivity and specificity of the multiplex PCR with culture on Ashdown’s agar

Finally, we chose to use this protocol to identify the presence of *B*. *pseudomallei* in natural soil samples from Thailand, and compare the results from our multiplex PCR assay with detection using the ‘gold standard’ culture on Ashdown’s agar. Rice field soil samples (n = 34) were collected from Ubon Ratchathani and Khon Kaen provinces, an endemic area of melioidosis in the Northeast of Thailand. All of the soil samples were cultured for *B*. *pseudomallei*, *B*. *thailandensis* and BTCV detection on Ashdown’s agar. Of the 34 samples, 12 were positive by both method and the multiplex PCR. Two of the remaining culture-negative samples were positive by multiplex PCR (Table 4). The remaining culture-negative samples were also negative when screened using the multiplex PCR. (Table 4). The calculated sensitivity and specificity of the multiplex assay are therefore 100% and 90.9%, respectively.

**Table 4:** Comparison between cultured-based method and *bimA* specific multiplex PCR to detect *B*. *pseudomallei* from 34 soil samples collected from endemic areas of melioidosis.

*bimA* specific PCR-based method	Culture-based method		Total
	Positive	Negative	
**Positive**	12	2	14
**Negative**	0	20	20
**Total**	12	22	34

## Discussion

The gold standard for detection of environmental *B*. *pseudomallei* and *B thailandensis* is based on bacterial culture from soil or water samples [44], which is a time-consuming process. In addition, the *B*. *pseudomallei* and *B thailandensis* variants BPBM [28] and BTCV [11], as well as *B*. *humptydooensis* [19] have all been shown to be present in the environment in melioidosis endemic areas. Severe disease is associated with *B*. *pseudomallei* and its BPBM variants, with some evidence of less fatal infections with the BTCV strains. Therefore, a simple technique to distinguish these three *Burkholderia* spp. is required for rapid and thorough epidemiological survey of these species in the environment, especially in Thailand where melioidosis is endemic and *B*. *thailandensis* and its variant strains (BTCV) are commonly isolated from soil and water.

In this study, we designed primers based on the known genetic variation of the *bimA* gene and used these in a multiplex PCR. This assay was able to detect and simultaneously discriminate between the DNA amplified from *B*. *pseudomallei* (936 bp), *B*. *thailandensis* (139 bp) and BPBM (586 bp). However, the assay could not differentiate *B*. *humptydooensis* from BPBM or *B*. *thailandensis* from BTCV. Despite enumerable soil and water surveys in the endemic areas, *B*. *humptydooensis* has only ever been isolated from a small and specific region of the Australian Northern territory [3] and has never been associated with disease in animals or humans. Therefore, we predict that any BPBM-like amplicon in our test is most likely to arise from the presence of a BPBM in the sample, rather than a *B*. *humptydooensis* strain.

Growing evidence supports the finding that BTCV strains can occasionally cause disease, usually non-fatal, in humans [7, 9] and therefore can be considered of pathogenic potential. Since the BTCV and *B*. *thailandensis* strains could not be differentiated by multiplex PCR, a second simplex PCR was designed based on the few single nucleotide sequence differences in the *B*. *thailandensis* and BTCV *bimA* genes (Fig 1B). As a result, only *B*. *thailandensis bimA* amplicons are generated in this singleplex PCR. Testing this simplex PCR against 10 strains of BTCV and 30 strains of *B*. *thailandensis* showed 100% accuracy in differentiation between *B*. *thailandensis* and BTCV. This study represents the first PCR-based method that allows the discrimination of *B*. *thailandensis* from BTCV strains. This is important since it is possible that the presence of the BTCV in some melioidosis endemic regions may confer protection against the development of melioidosis. Little is known about the prevalence of BTCV and its impact on *B*. *pseudomallei* infection. Thus, the combined use of our multiplex and simplex PCRs described in this study will be useful to survey the presence of BTCV in both non-endemic and endemic areas, with the aim of understanding the genetic diversity, virulence and evolution of these emerging organisms.

An additional key advantage of the *bimA*-based multiplex PCR assay is not only to detect *B*. *pseudomallei*, *B thailandensis* and the BTCV, but also to discriminate *B*. *pseudomallei* from BPBM in a single multiplex PCR reaction. BPBM strains have been associated with neurologic melioidosis, which is a serious and potentially fatal form of *B*. *pseudomallei* infection [22, 57]. In an animal model, BPBM were more virulent when delivered intranasally or subcutaneously than typical *B*. *pseudomallei* isolates [22]. To date, BPBM strains have not been isolated from the environment in Southeast Asia, however it is possible that it is present but has been mis-identified as *B*. *pseudomallei*. Therefore, the possibility that this variant strain is present in a wider geographical area including Thailand cannot be excluded.

By spiking soil samples with known numbers of viable *B*. *pseudomallei*, *B*. *thailandensis* and BPBM, we were able to ascertain that the sensitivity of our multiplex PCR assay to be 5–6 CFU/g soil sample. Although the sensitivity of this assay is not as high as a previously reported singleplex PCR to detect *B*. *pseudomallei* (1–1.5 CFU/g of soil sample) [58], our multiplex PCR assay could simultaneously detect the *Burkholderia* species of most clinical importance. An added benefit of our *bimA*-based PCR method is that the readout is easy to interpret, and unlikely to be interpreted incorrectly, since it relies on significant differences in amplicon size (936 vs 586 vs 139 bp). Furthermore, using natural soil samples we were able to compare the sensitivity and specificity of the multiplex assay with the existing conventional bacterial culture method [20, 59]. Multiplex PCR to detect *B*. *pseudomallei* from 34 soil samples revealed that the sensitivity and specificity of the multiplex PCR, in comparison with culture on Ashdown’s agar, are 100% and 90.9% respectively, suggesting that it is a reliable alternative method.

Although the sample size is small, the soil samples were collected from 2 different provinces which approximately 282 km apart which were expected to contain different bacterial populations and differ in major soil nutrients. In addition, *Burkholderia* spp. included in this study were composed of multiple different ST and were collected from Thailand and Australia, the endemic area of melioidosis.

In conclusion, we report herein sets of PCR primers that can be used in a combined multiplex and singleplex PCR-based way to detect *B*. *pseudomallei* and *B*. *thailandensis*, BPBM and BTCV in environmental samples. The multi-species differentiation assay using *bimA*-based multiplex PCR technique presented here is a simple, specific, and sensitive technique that will be useful for environmental sampling study and for prediction of areas of increased risk of disease in humans and animals.

## Supporting information

S1 Fig Agarose gel electrophoresis of *bimA* DNA fragments amplified from 10 isolates of BTCV. (A) Multiplex PCR of 10 BTCV strains including strains E555, SBXPR001, SBXSR007, SBXPL001, SBXPL015, SBXRY031, SBXPR001, SBXCC001, SBXCC003 and WBXUBA33005104 (lanes 1–10) with multiplex PCR primers (BimA_Bps_-F/BimA_com-_R, Bim_BPBM_-F/BimA_com-_R, and BimA_Bth_-F/BimA_Bth-_R). The expected 139-bp DNA fragments were detected in all samples. (B) Singleplex PCR of BTCV strains from (A) amplified with primers BimA_Bth_-SF and BimA_Bth-_R primers (lanes 1–10). Lanes P (mixture of *Burkholderia* spp. genomic DNA) and N (distilled water) are positive and negative controls, respectively. Lane M is 100 bp DNA ladder.
(TIFF)

S2 Fig Multiple nucleotide sequence alignment of *bimA* gene among of BPBM (MSHR491), *B*. *mallei* (ATCC23344) and *B*. *humptydooensis* (MSMB43, MSMB1588). The nucleotide sequences in red correspond to the Bim_BPBM_ and Bim_com_ primers.
(TIFF)
