# Supplementary figures and images for: Detection and differentiation of Burkholderia species with pathogenic potential in environmental soil samples
Source: PLoS One. 2021 Jan 7;16(1):e0245175. doi: 10.1371/journal.pone.0245175 (PMC7790303; doi:10.1371/journal.pone.0245175)

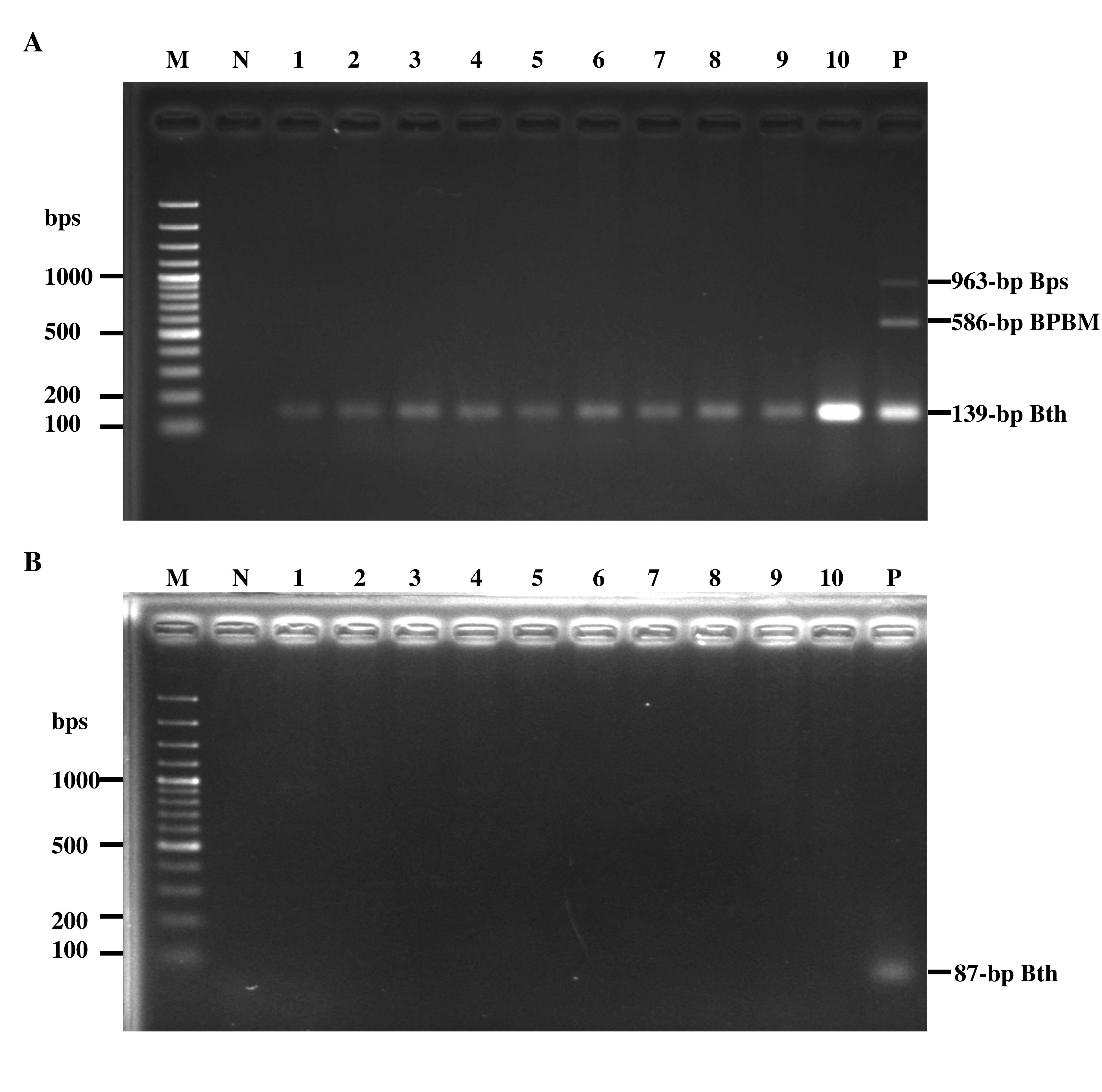

Supplement: S1 Fig — (A) Multiplex PCR of 10 BTCV strains including strains E555, SBXPR001, SBXSR007, SBXPL001, SBXPL015, SBXRY031, SBXPR001, SBXCC001, SBXCC003 and WBXUBA33005104 (lanes 1–10) with multiplex PCR primers (BimABps-F/BimAcom-R, BimBPBM-F/BimAcom-R, and BimABth-F/BimABth-R). The expected 139-bp DNA fragments were detected in all samples. (B) Singleplex PCR of BTCV strains from (A) amplified with primers BimABth-SF and BimABth-R primers (lanes 1–10). Lanes P (mixture of Burkholderia spp. genomic DNA) and N (distilled water) are positive and negative controls, respectively. Lane M is 100 bp DNA ladder. (TIFF) [file pone.0245175.s001.tiff]

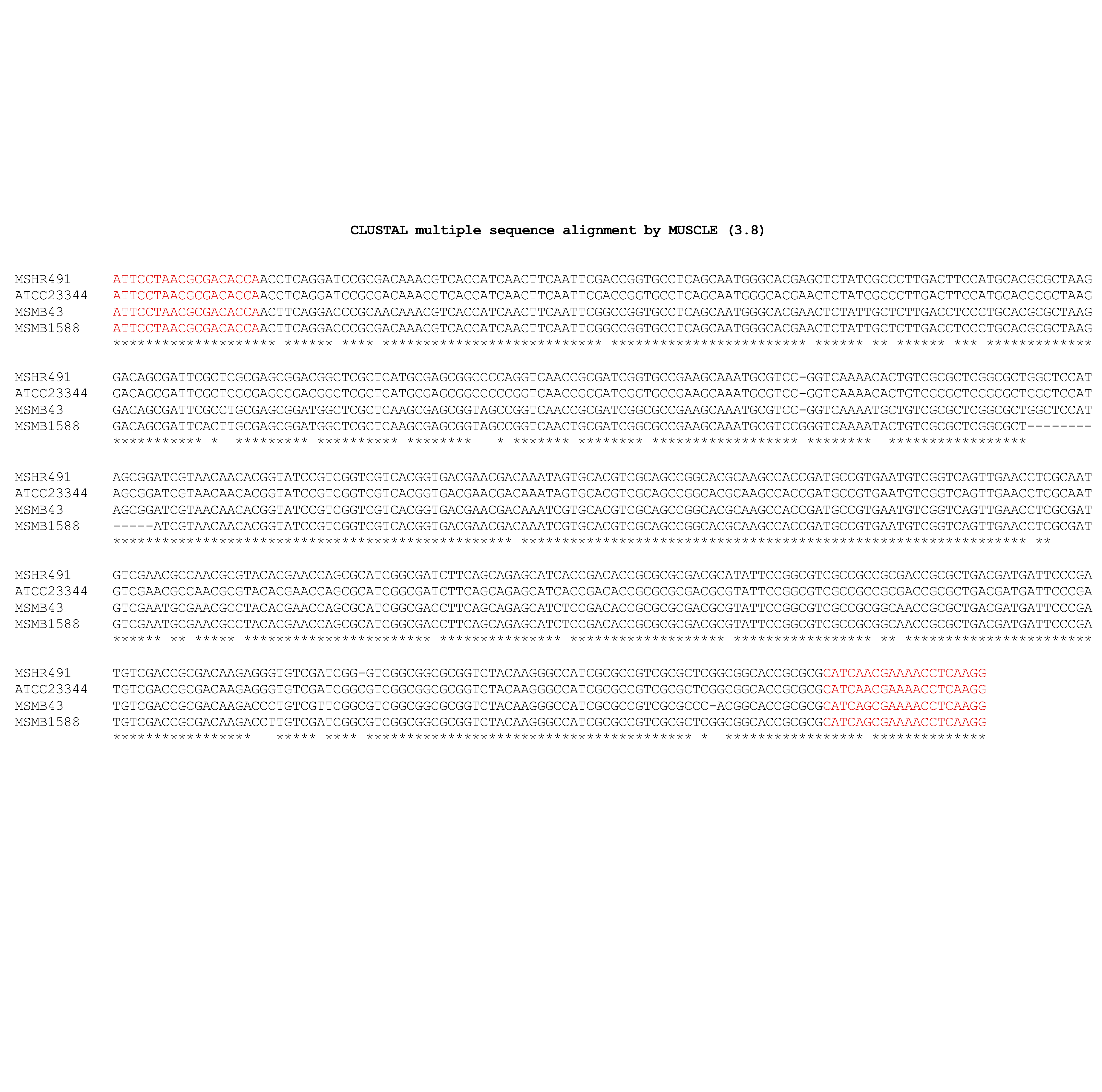

Supplement: S2 Fig — The nucleotide sequences in red correspond to the BimBPBM and Bimcom primers. (TIFF) [file pone.0245175.s002.tiff]
